# Planar polarized Rab35 functions as an oscillatory ratchet during cell intercalation in the *Drosophila* epithelium

**DOI:** 10.1038/s41467-017-00553-0

**Published:** 2017-09-07

**Authors:** Cayla E. Jewett, Timothy E. Vanderleest, Hui Miao, Yi Xie, Roopa Madhu, Dinah Loerke, J. Todd Blankenship

**Affiliations:** 1 Department of Biological Sciences, University of Denver, Denver, CO 80208 USA; 2 Department of Physics, University of Denver, Denver, CO 80208 USA

## Abstract

The coordination between membrane trafficking and actomyosin networks is essential to the regulation of cell and tissue shape. Here, we examine Rab protein distributions during *Drosophila* epithelial tissue remodeling and show that Rab35 is dynamically planar polarized. Rab35 compartments are enriched at contractile interfaces of intercalating cells and provide the first evidence of interfacial monopolarity. When Rab35 function is disrupted, apical area oscillations still occur and contractile steps are observed. However, contractions are followed by reversals and interfaces fail to shorten, demonstrating that Rab35 functions as a ratchet ensuring unidirectional movement. Although actomyosin forces have been thought to drive interface contraction, initiation of Rab35 compartments does not require Myosin II function. However, Rab35 compartments do not terminate and continue to grow into large elongated structures following actomyosin disruption. Finally, Rab35 represents a common contractile cell-shaping mechanism, as mesoderm invagination fails in Rab35 compromised embryos and Rab35 localizes to constricting surfaces.

## Abstract (web summary)

Various stages of tissue morphogenesis involve the contraction of epithelial surfaces. Here, the authors identify the Rab GTPase Rab35 as an essential component of this contractile process, which functions as a membrane ratchet to ensure unidirectional movement of intercalating cells.

## Introduction

How do molecular, cell, and developmental processes drive the production of stereotypical tissue forms and body shapes? This has been a central question in biology, and the pursuit of such answers has driven key discoveries in understanding how cell assemblies coordinate cellular adhesion to enable multicellular life. A common characteristic of many higher organisms is an elongated body axis. This elongated body plan, often oriented along the anterior–posterior (AP) axis, is frequently reiterated at the organ level. Cell intercalation is one of the primary mechanisms that is utilized to direct tissue elongation^1^. Tissue elongation is essential to the shaping of an elongated body axis^1, 2^, as well as the development of many internal organs, such as the palate, cochlea, gut, and kidney^3–6^.

In epithelial sheets, processes that drive cells to change topological relationships can be harnessed by developmental processes to effect changes in tissue architecture^7^. The oriented contraction of T1 (or vertical, AP) interfaces followed by the growth of T3 interfaces leads to tissue narrowing and extension. This cellular reshaping requires the function of apical and junctional cytoskeletal and adhesion proteins^8–13^. Interestingly, force generation and changes in cell shape are not continuous during cell intercalation, but instead occur in pulses with intervening stable periods. In one model, pulsatile actomyosin forces generated in the apical/medial cell regions are believed to initiate intercalary movements through oscillations in apical cell areas^14–16^. Subsequent enrichment of Myosin II at adherens junctions leads to higher tensile forces at AP interfaces and the destabilization of E-cadherin adhesive complexes^9, 16–19^. There is thus a system of planar polarized protein distributions during cell intercalation, with F-actin and Myosin II enrichments at AP interfaces and apical domains, and adherens junction-associated proteins such as Bazooka/Par-3, E-cadherin, and Armadillo/ß-catenin enriched at dorsal and ventral (DV) neighboring interfaces^8–10^. This combination of tension-producing actomyosin networks and cadherin-dependent adhesion complexes are believed to be central determinants directing early morphogenesis in the Drosophila embryo; however, the role of membrane trafficking in guiding these events has been less clear^20, 21^. Additionally, how periods of active cytoskeletal contraction are tied to processes that function at the plasma membrane to ensure the consolidation and irreversibility of changes requires clarification.

The Rab family of small GTPase proteins is key mediators of membrane trafficking and cytoskeletal dynamics. Rab proteins regulate membrane compartment behaviors through their association with tethering and trafficking effectors^22–24^, and mutations in Rab proteins are associated with a variety of diseases and developmental disorders^25–28^. The Rab trafficking pathways that operate during cell intercalation in the early *Drosophila* gastrula have remained undefined, although the function of classic Clathrin and Dynamin-dependent early endocytic pathways has been explored^18^. Indeed, it has been demonstrated that Formin and Myosin II proteins direct the endocytic uptake of dextran through specialized CIV (cortical immobile vesicle) structures^18^. Additionally, in *Drosophila*, Rab35 has been shown to play a critical role in directing the morphogenesis of tracheal tube growth as well as synaptic vesicle sorting at the neuromuscular junction^29, 30^. In tissue culture models, Rab35 functions early in endosomal pathways to drive the generation of newborn endosomes and is essential for the terminal steps of cytokinesis and neurite outgrowth^31–38^.

Here, we show that Rab35 demonstrates remarkable compartmental behaviors at the plasma membrane. Rab35 compartments are initially contiguous with the cell surface and form dynamic structures that grow and shrink on the minute time scale. In the absence of Rab35 function, cell interfaces undergo contractile steps, but these steps rapidly reverse themselves, consistent with Rab35 mediating an essential “ratcheting” function that directs progressive interface contraction. Rab35 compartments form independently of Myosin II function, but require actomyosin forces to terminate. These compartments further function as endocytic hubs that have transient interactions late in their formation with Rab5 and Rab11 endosomes.

## Results

### Rab family protein localization during cell intercalation

During *Drosophila* germband extension (GBE), cells undergo the coordinated planar contraction of interfaces between AP neighboring cells^2, 9, 10^. Actomyosin force generating networks in the apical/medial and junctional cell regions demonstrate periodic contractile behaviors and direct intercalary movements^9–12, 15, 16, 18, 19, 39^. However, the trafficking networks that are functioning in these cells, and the degree to which their activities may be coordinated with cytoskeletal forces that control plasma membrane topologies, have been unclear. To identify the relevant trafficking pathways in epithelial cells undergoing cell intercalation, we focused on the behaviors of Rab proteins in vivo by screening the expression and localization of the 31 *Drosophila* Rab proteins as driven by Rab-Gal4 knock-ins^40, 41^. We found that eight Rab proteins (Rab 4, 5, 8, 11, 14, 23, 35, 39) were expressed at levels significant enough to be observed by live imaging (Supplementary Fig. 1). Of these, Rab35 had the most intriguing behaviors that were associated at or near the plasma membrane.

### Rab35 behaviors are enhanced at AP interfaces

**Fig. 1:**
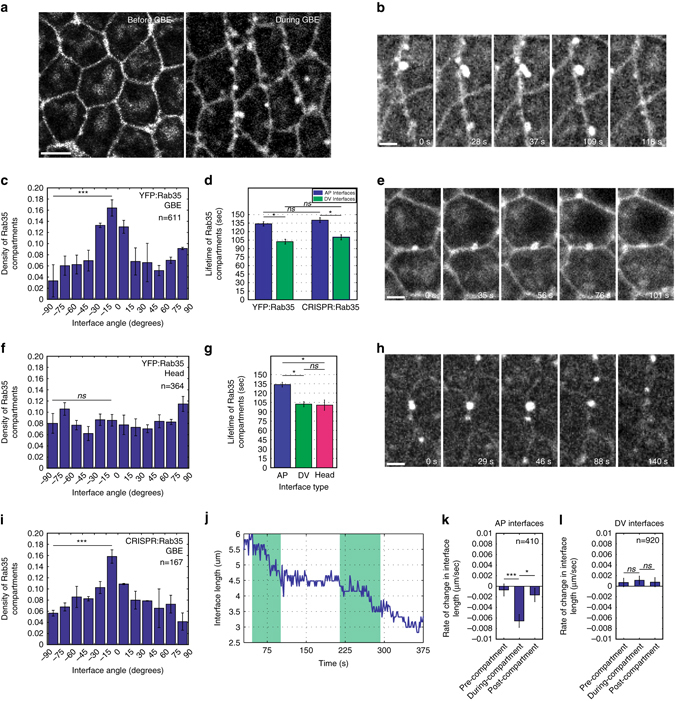
Planar polarized Rab35 compartments associate with AP interfaces during periods of active interface contraction. **a**, **b** Time-lapse images of embryo expressing YFP:Rab35. All images, anterior is to the *left* and ventral is *down*. **a** YFP:Rab35 compartments localize to AP cell interfaces after GBE begins (*right image*). **b** Representative images depicting the life cycle of a Rab35 compartment: initiation of YFP:Rab35 compartment (0 s), growth phase (28 s), maximum compartment size (37 s), shrinkage (109 s), and compartment termination (118 s). **c** Rab35 compartments occur more frequently at AP interfaces. Compartment density plotted as a function of binned interfaces angles in the germband (*n* = 611 compartments). **d** YFP:Rab35 compartment lifetimes are longer at AP interfaces (*n* = 205 compartments). Endogenous CRISPR:GFP:Rab35 displays similar lifetimes to YFP:Rab35 (*n* = 170 compartments). **e**–**g** YFP:Rab35 compartments in the non-intercalating head region (**e**) show an absence of planar polarity (**f**) and lifetimes comparable to DV-localized Rab35 (**g**) (*n* = 364 (**f**) and 198 (**g**) compartments). **h**, **i** At endogenous levels, CRISPR:GFP:Rab35 demonstrates similar compartmental dynamics (**h**) and planar polarity (**i**) to YFP:Rab35, although non-compartmental plasma membrane localization of Rab35 is reduced (**h**) (*n* = 167 compartments). **j** Rab35 compartments are associated with periods of interface contraction. Automated segmentation analysis and compartment recognition showing a plot of interface length over time with association of YFP:Rab35 compartments shaded in *green*. **k**, **l** Decreases in AP interface length strongly correlate with the presence of Rab35 compartments. Rate of change in interface length 30 s before initiation of Rab35 compartment (pre-compartment), during the lifetime of a Rab35 compartment (during-compartment), and 30 s after termination of a Rab35 compartment (post-compartment). AP interfaces show significant decreases in interface length during the lifetime of Rab35 compartments (**k**), whereas DV interfaces show no significant changes in interface length (**l**). Negative values indicate contractions in interface length. Student’s *t*-test, *P < 0.05, **P < 0.005, ***P < 0.0005. *Error bars* indicate standard error. *Scale bar* in **a** is 5 microns. *Scale bars* in **b**, **e**, **h** are 2.5 microns

Rab35, as shown by a YFP:Rab35 transgene, displays striking compartmental behaviors at interfaces between AP neighboring cells during cell intercalation (Fig. 1a, b; Supplementary Movie 1). Prior to cell intercalation, Rab35 is present at low levels at the plasma membrane (Fig. 1a). However, once GBE has initiated, spherical and tubular Rab35 compartments form at the plasma membrane, grow for 1–2 min, and then terminate and disappear from the plasma membrane (Fig. 1a, b). Rab35 compartmental behaviors are planar polarized, with 57% of measured compartments present at interfaces ranging within 30˚ of the vertical axis (AP interfaces), while only 11% of compartments were present at interfaces within 30˚ of the horizontal axis (DV interfaces) (Fig. 1c). Compartmental behaviors are longer-lived at vertical interfaces with an average lifetime of 135 s, while less frequent, shorter-lived compartments are apparent at horizontal interfaces (104 s, Fig. 1d). Compartments grow for ~60% of the total compartmental time, before rapidly resolving and disappearing from view. In the head region, where cell intercalation does not occur, Rab35 compartments are less numerous, not planar polarized, and have shorter lifetimes that are similar to those observed at non-contracting DV interfaces (Fig. 1e–g).

To examine the behavior of endogenous Rab35, we utilized CRISPR/Cas9 technology to tag Rab35 with an N-terminal green fluorescent protein (GFP) at the genomic locus. Insertion at the genomic locus was confirmed by genomic polymerase chain reaction (PCR) (Supplementary Fig. 2). This endogenous GFP:Rab35 is homozygous viable and fertile, indicating that CRISPR GFP:Rab35 is fully functional. Endogenous GFP:Rab35 localization is similar to YFP:Rab35 expression, although non-compartmental Rab35 localization at the plasma membrane is largely lost (Fig. 1h). The planar polarized behaviors of Rab35 compartments are identical between the two backgrounds, and no significant difference was observed in compartmental lifetimes (Fig. 1d, i).

### Rab35 compartments are associated with contractile steps

We then examined the relationship between Rab35 compartments and changes in interface length. Automated segmentation and tracking of individual interface lengths showed that Rab35 compartments are primarily present during periods of interface contraction (Fig. 1j). However, only at AP interfaces are Rab35 compartmental behaviors correlated with periods of interface contraction (Fig. 1k). At DV interfaces, no significant difference in changes of interface length is observed regardless of whether a Rab35 compartment is present (Fig. 1l).

### Rab35 is required for progressive interface contraction

**Fig. 2:**
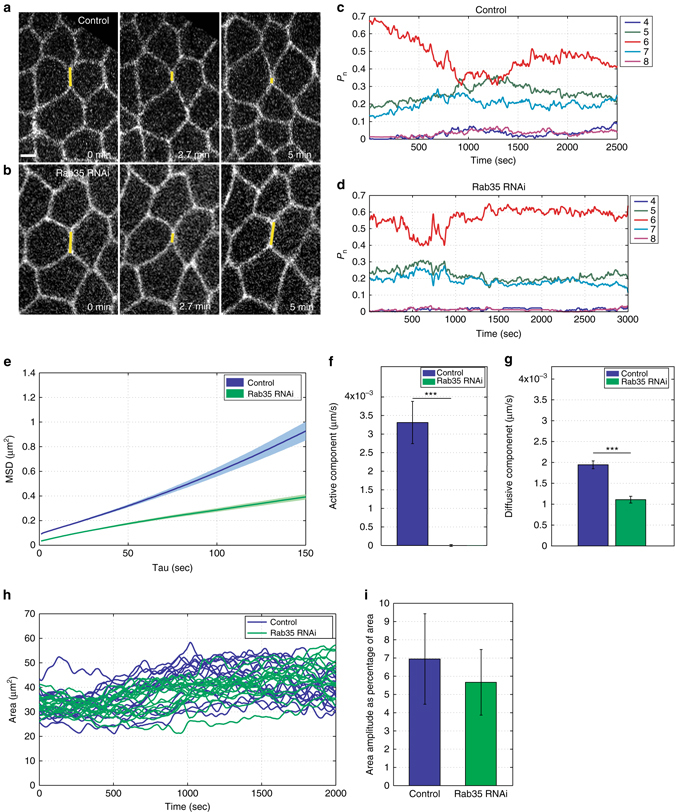
Rab35 promotes progressive interface contraction required for cell intercalation. **a**, **b** Still frames of embryos expressing a cell outline marker (Resille:GFP) injected with control dsRNA (Rhodopsin3, Rh3, **a**) or Rab35 dsRNA (**b**). In control-injected embryo, the AP interface (*yellow line*) has fully contracted after 5 min. **b** In the Rab35 knockdown embryo, the AP interface (*yellow line*) undergoes an initial contraction (*middle panel*) but reverses itself. After 5 min the interface shows no net contraction (*right panel*). **c**, **d** Frequency of *n*-sided cells during germband extension in control Rh3 dsRNA and Rab35 dsRNA embryos (*n* = 74 and 83 cells, respectively). Germband extension initiates as a primarily hexagonal lattice in both control and Rab35 dsRNA embryos. In control embryos, the number of six-sided cells rapidly decreases, while the number of alternatively sided cells increases as convergent extension movements occur. In Rab35 dsRNA embryos, there is a slight initial decrease in hexagonal cell number, before cell topologies return to the initial configuration. **e**–**g** Rab35 dsRNA embryos have a decrease in productive interface contractions with reduced MSDs (**e**) and a near absence of the active MSD component (**f**) and decreased diffusive component (**g**). *n* = 286 (control) and 139 (Rab35 dsRNA) cells. **h**, **i** Oscillations in apical area still occur in Rab35 dsRNA embryos (**h**), with an amplitude similar to control Rh3 dsRNA-injected embryos (**i**). *n* = 631 (control) and 340 (Rab35 dsRNA) cells. Student’s *t*-test, ***P < 0.0005. *Error bars* indicate standard error. *Scale bar* is 2.5 microns

As Rab35 compartmental behaviors are correlated with periods of interface contraction, we next examined the effect of disrupting Rab35 function on cell topologies and neighbor relationships during GBE. In Rab35 compromised embryos, cell intercalation was deeply disrupted (Fig. 2a, b). The epithelial sheet maintains a primarily hexagonal configuration, with little evidence of neighbor exchange (Rab35 dsRNA or CRISPR GFP:Rab35; GFP shRNAi embryos, Fig. 2a–d; Supplementary Fig. 3).

We therefore examined the early phase of cell intercalation, when cells are undergoing T1–T2 transitions and rosette formation occurs^9, 10^. We found that the overall contraction of AP interfaces was deeply compromised, with decreased net displacements and active components (the systematic change of interface length that scales linearly with time, see “Methods”) in Rab35 dsRNA-injected embryos (Fig. 2e–g). However, when individual AP interfaces were examined in Rab35-disrupted embryos, wobble-like behaviors were often observed in which initial periods of interface shortening occurred, but were then followed by a re-lengthening of the interface (Fig. 2b; Supplementary Movie 2). Oscillations in apical area still occur, and the amplitude of these oscillations is similar to that observed in control-injected embryos (Fig. 2h, i).

**Fig. 3:**
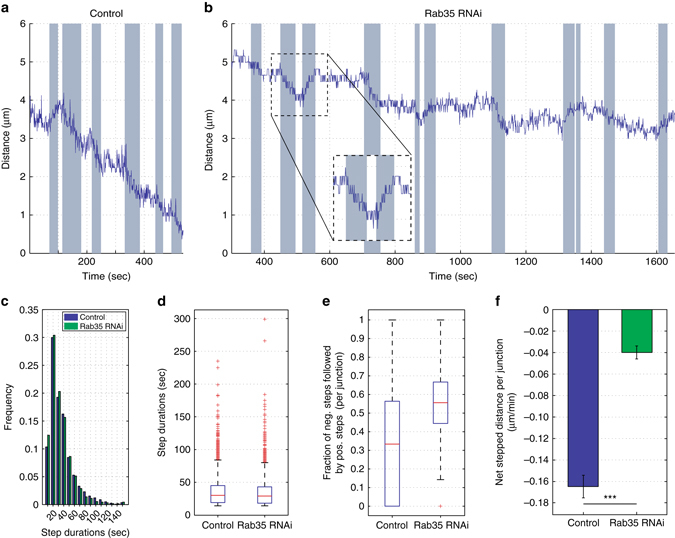
Rab35 performs a ratcheting function during interface contraction. **a**, **b** Individual AP interface length plots in control Rh3 (**a**) and Rab35 dsRNA (**b**) embryos. Automated step detection indicates periods of active interface length change (*shaded regions*). A control interface progressively contracts within 8 min (**a**), while a Rab35 dsRNA interface undergoes active contractile steps followed by lengthening reversals (**b**, *inset*). After nearly 30 min, the AP interface is still preserved in Rab35 dsRNA embryo. **c**, **d** The frequency (**c**) and duration (**d**) of active stepping is similar in control and Rab35 dsRNA embryos. *n* = 2990 (control) and 2536 (Rab35 dsRNA) steps. **e**, **f** Contractile steps are often reversed by positive stepping in Rab35 dsRNA embryos (**e**) producing a decrease in net displacement (**f**). *n* = 328 (control) and 340 (Rab35 dsRNA) interfaces. *Error bars* indicate standard error. Student’s *t*-test, ***P < 0.0005

In order to examine the wobble-like behaviors in Rab35-disrupted embryos more closely, we used an automated step-detection algorithm to detect periods of directed movement. Intriguingly, AP interfaces in Rab35 compromised embryos still undergo steps with the same frequency, duration, and size as in control embryos (Fig. 3a–d). However, contractile steps in Rab35-disrupted embryos are often followed by steps with a positive displacement (Fig. 3b *inset*, e). Due to these reversals, the net *stepped* distance is much lower in Rab35 compromised embryos (Fig. 3f). These results are consistent with Rab35 functioning as a ratcheting module that directs progressive shortening of AP interfaces in response to apical area oscillations.

### AP patterning re-directs Rab35 compartment formation

**Fig. 4:**
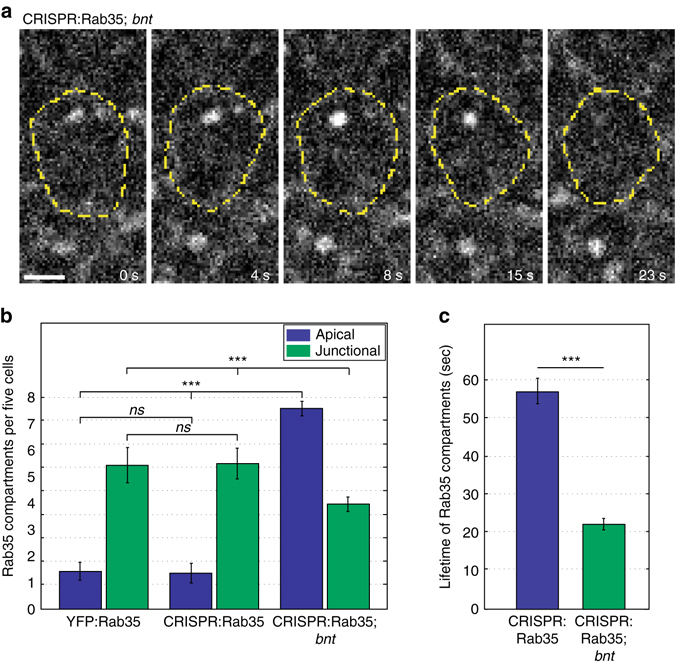
The Rab35 ratchet is engaged by AP patterning. **a**–**c** Rab35 compartments localize apically away from cell interfaces and possess reduced lifetimes in embryos lacking AP patterning (*bicoid nanos torsolike*, *bnt*, mutant embryos). **a** Rapid Rab35 compartmental formation and termination at the apical surface in *bnt* embryos (cell outline marked in *yellow*). **b**, **c** Majority of Rab35 compartments form apically (**b**) and possess decreased lifetimes (total lifetimes of apical and interfacial compartments, **c**) in *bnt* mutant embryos. *n* = 681 (YFP:Rab35), 454 (CRISPR:GFP:Rab35), and 782 (CRISPR:GFP:Rab35; *bnt*) compartments. Mann–Whitney U Test, ***P < 0.0005. *Error bars* indicate standard error. *Scale bar* is 2.5 microns

The AP patterning system is also required to direct and engage the Rab35 ratchet at individual interfaces. In embryos that lack all AP patterning (*bicoid nanos torso-like* mutant embryos), the majority of Rab35 compartments form on the apical surface, and not at interfaces (Fig. 4a, b). These compartments possess much shorter lifetimes than those that form in wild-type embryos (Fig. 4c), revealing that the AP patterning system lengthens Rab35 compartmental behaviors and directs them to contracting interfaces.

### Actomyosin function is required for compartment termination

**Fig. 5:**
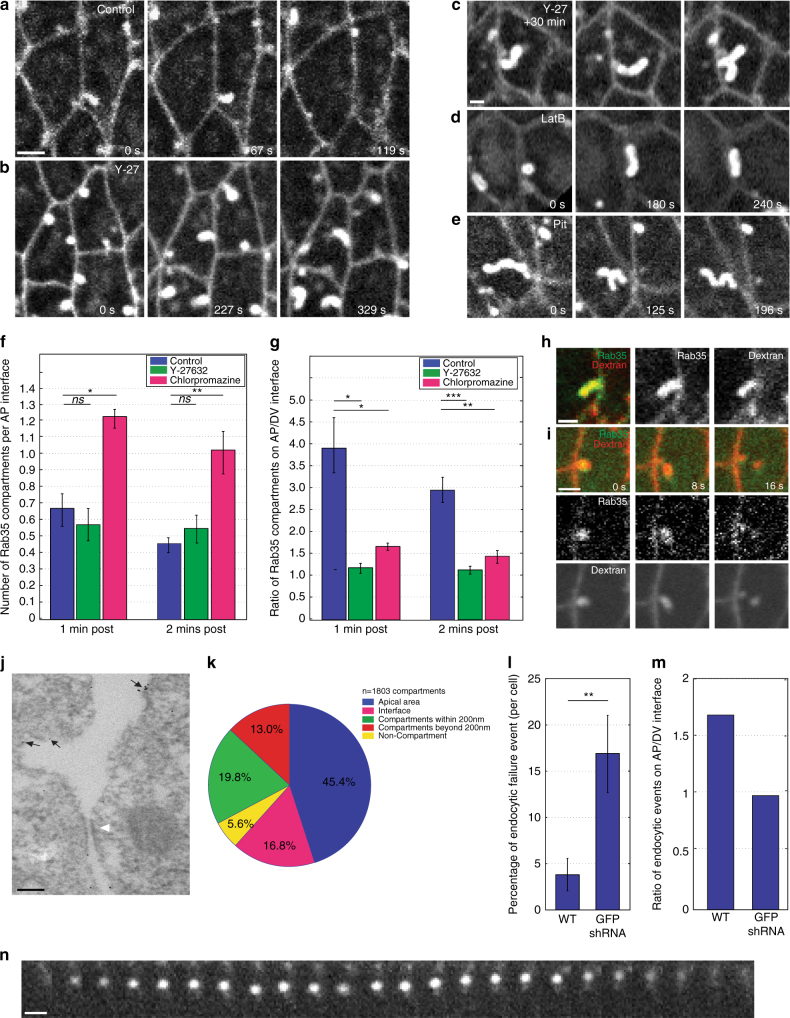
Rab35 compartments form independently but require actomyosin and endocytic pathways for termination and polarity. **a**–**c** YFP:Rab35-labeled embryo injected with water control (**a**) or Y-27632 Rok inhibitor (**b**), during cell intercalation. Compartments form and resolve within 119 s in control embryo (**a**), whereas compartments form, but then fail to resolve, in Y-27632 embryo after 329 s (**b**). **c** Embryo 30 min post Y-27632 injection, showing elongation and bifurcation of Rab35 compartments. **d** Rab35 compartments form independently of F-actin function, but fail to terminate in LatB-injected embryos. Note compartment growth along plasma membrane. **e** Compartments grow into long, tubular structures and do not terminate in a YFP:Rab35 embryo injected with the endocytic inhibitor Pitstop2. **f** The number of Rab35 compartments per AP interface in water control, Y-27632, and chlorpromazine-injected embryos. Additional Rab35 compartments are present 1 and 2 min post-chlorpromazine injection. **g** Planar polarity of Rab35 compartments is compromised in Y-27632 and chlorpromazine-injected embryos, as opposed to water control. *n* = 133 (water), 156 (Y-27632), and 128 (chlorpromazine) compartments. **h**, **i** Rab35 compartments are contiguous with the cell surface. Rab35 compartments are labeled immediately when rhodamine-labeled dextran is injected extracellularly (**h**), before scissioning from plasma membrane (**i**) or shedding cytoplasmic dextran-labeled compartments (Supplementary Fig. 4f, g) in a YFP:Rab35 embryo. **j** Immunogold TEM images of anti-GFP CRISPR:GFP:Rab35 reveals Rab35 (*arrows*) is present in plasma membrane infoldings that are contiguous to the interface and present near adherens junctions (*white arrowhead*). **k** Quantification of Rab35 immunogold localization (*n* = 1803 compartments). **l** The number of failed endocytic events quadruples in Rab35-disrupted embryos. *n* = 159 (WT) and 264 (GFP shRNA) events. **m** The enhancement of endocytic rates at AP interfaces is lost in Rab35-disrupted embryos. *n* = 123 (WT) and 170 (GFP shRNA) events. **n** Kymograph of failed endocytic event: a dextran-positive structure begins to form, but then reverses and collapses back into the plasma membrane (panel every 2 s). Student’s *t*-test, *P < 0.05, **P < 0.005, ***P < 0.0005. *Error bars* indicate standard error. *Scale bar* in **a** is 2.5 microns, in **c**, **h**, **i** 1.25 microns, and 100 nm in **j**

Since actomyosin forces have been shown to direct both interface contraction as well as apical area oscillation^9, 10, 15, 16, 19, 39^, we examined the relationship between Rab35 behaviors and Myosin II function. Embryos injected with Y-27632, an inhibitor of the Myosin II activator Rho kinase (Rok), displayed remarkable Rab35 compartmental behaviors (Fig. 5a–c). Initiation of Rab35 compartments still occurred in Y-27632 embryos; however, Rab35 compartments never resolved and continued growing without termination (Fig. 5b, c). This produced compartments that could span entire cell diameters, at times bifurcating and displaying striking dynamics (Supplementary Movie 3). These results were confirmed by examining embryos mutant for the *Drosophila* Myosin Regulatory Light Chain, *spaghetti squash* (*sqh*, Supplementary Fig. 4a). Acute disruption of F-actin function also showed that Rab35 compartments do not terminate in embryos injected with Latrunculin B (Fig. 5d). Unlike Y-27632 injection, elongated compartments in LatB-injected embryos often aligned with the plasma membrane, suggesting that F-actin-driven cortical stiffness is required for compartments to project internally into the cell.

Given these results, we imaged the behaviors of Rab35 and Myosin II. YFP:Rab35 compartments initiated without appreciable Myosin II localization (Supplementary Fig. 4e), consistent with Rab35 behaviors observed in Myosin II compromised embryos. In the second half of a Rab35 compartment’s lifetime, significant mCh:Myosin II signal became apparent, peaking with the termination of the compartment. The correlation of Rab35 behaviors with periods of interface contraction, as well as the appearance of Rab35 prior to Myosin II and the failure of Rab35 compartments to terminate when Myosin II function is compromised, suggests a model in which Rab35 promotes interfacial ratcheting by taking up membrane in specialized compartments that are then terminated by junctional Myosin II activity. It is also interesting to note that while Rab35 compartment formation is independent of Myosin II activity, Rab35 planar polarity is disrupted in Y-27632-injected embryos, indicating that apical/medial Myosin II flows may be necessary for planar engagement of Rab35 behaviors (Fig. 5g).

### Rab35 compartments act as endocytic hubs

What then happens to membrane in Rab35 compartments and how does Myosin II activity lead to compartment termination? Previous work on contracting interfaces demonstrated the existence of membranous CIVs that serve as hubs of clathrin-mediated endocytosis^18^. We therefore examined Rab35 compartmental behaviors in the presence of two different endocytic inhibitors, as well as in *shibire* ^*ts*^ (the *Drosophila* Dynamin) alleles. Treating embryos with either PitStop2 or chlorpromazine inhibited interface contraction and produced Rab35 compartments that, similar to Y-27632-injected embryos, could not terminate (Fig. 5e, Supplementary Fig. 4b, d). *shibire* ^*ts1*^; GFP:Rab35 embryos at the non-permissive temperature (31 °C) also produced stable, elongated Rab35 tubules (Supplementary Fig. 4c). Endocytically compromised embryos also possessed an increased number of Rab35 compartments at AP interfaces, as well as defects in Rab35 planar polarity (Fig. 5f, g).

We then determined if Rab35 compartments are contiguous with the cell surface by injecting fluorescently labeled dextran into the perivitelline space. Rab35 compartments immediately filled with dextran (Fig. 5h, i), indicating that they are open to the extracellular space and contiguous with the plasma membrane. Interestingly, these compartments appear to have a privileged composition, excluding some common markers of the plasma membrane (Gap43:mCh, Spider:GFP), while highlighted by PLC-PH-GFP (a sensor for the plasma membrane PIP_2_ species PtdIns(4,5)P_2_, Supplementary Fig. 4i). Additionally, dextran-marked vesicles can be observed leaving Rab35 compartments, consistent with endocytosis occurring from these structures (Fig. 5i, Supplementary Fig. 4f, g). Ultrastructural analysis of Rab35 compartments through immunogold transmission electron microscopy (TEM) shows the majority of Rab35-associated gold particles are located at or near the plasma membrane, in the same regions marked by fluorescently tagged Rab35 (Fig. 5j, k; Supplementary Fig. 4h). Importantly, Rab35 is often found marking open tubular compartments located near adherens junction (Fig. 5j, k). These results demonstrate that Rab35 compartments form as infoldings of the plasma membrane that drive interface shortening. Rab35 compartmental behaviors are then terminated through Myosin II activity and endocytic processes.

We then examined endocytic events in Rab35 compromised embryos (Fig. 5l, n). Previous work has shown that endocytic rates are enhanced at AP interfaces as compared to DV interfaces^18^, a finding that we also observe (Fig. 5m). However, in Rab35-disrupted embryos there is an equal probability of endocytic events at AP or DV interfaces (Fig. 5m). We also found that there was a four-fold increase in the number of failed endocytic events in Rab35-disrupted embryos (Fig. 5l, n), suggesting that Rab35 compartments may serve to direct the efficient and enhanced uptake of materials from AP interfaces.

### Rab35 directs plasma membrane into endosomal compartments

**Fig. 6:**
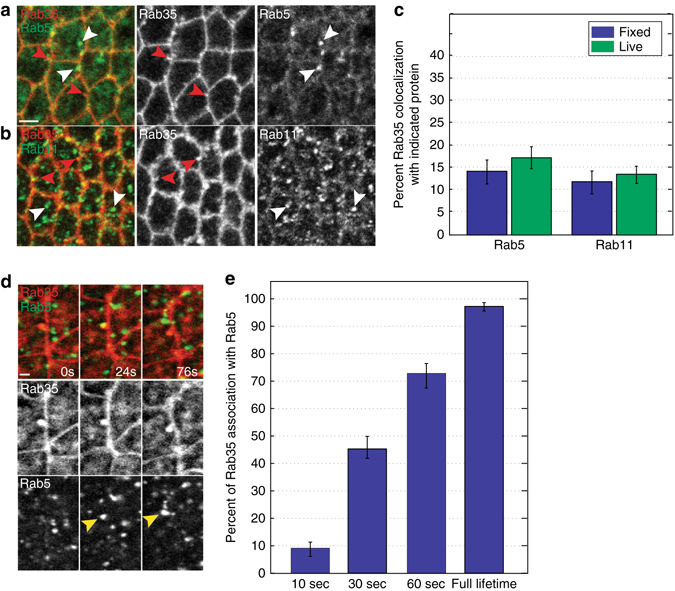
Rab35 compartments direct endocytic membrane to endosomes. **a**, **b** Rab35 has a low degree of colocalization with Rab5 (**a**, *green*) or Rab11 (**b**, *green*) in fixed tissue by immunofluorescence. *White arrowheads* indicate typical Rab5 (**a**) or Rab11 (**b**) compartments, while *red arrowheads* indicate interfacial Rab35 compartments (**a**, **b**). **c** Quantification of colocalization in **a**, **b**. *n* = 210 (fixed, with Rab5), 198 (live, with Rab5), 245 (fixed, with Rab11), and 259 (live, with Rab11) compartments. **d** Live imaging of YFP:Rab5 (*green*) and mCh:Rab35 (*red*). An AP Rab35 compartment (0 s) displays no association with Rab5. Later in the lifetime of the Rab35 compartment (24 s) a Rab5 early endosome associates with the compartment (*yellow arrowhead*) and grows in size before separating (76 s). **e** Quantitation of association between Rab35 compartments and Rab5. Rab35 compartments are increasingly likely to associate with early endosomal Rab5 as they progress toward the end of their lifetimes. *n* = 197 compartments. *Error bars* indicate standard error. *Scale bars* are 2.5 microns in **a** and 1.25 microns in **d**

If Rab35 guides the movement of AP interface-associated plasma membrane and proteins into endocytic pathways, we asked if we could observe an interaction between Rab35 compartments at the cell surface and either early or recycling endosomes. Examining fixed tissues, we observed little colocalization between Rab5 (early endosomes) and Rab11 (recycling endosomes) (Fig. 6a–c). However, there was a small fraction (<15%) of Rab35 compartments that reproducibly displayed a partial colocalization with endosomal compartments. We therefore wondered if these compartments might represent a transient interaction between Rab35 and endosomes, and analyzed rapid live imaging acquisitions from either YFP:Rab5; mCh:Rab35 or YFP:Rab11; mCh:Rab35-expressing embryos. Although no pattern emerged from observing YFP:Rab11; mCh:Rab35 embryos, Rab5 displayed interesting behaviors with Rab35 (Fig. 6d, e). Rab35 compartments in the early stages of their lifetimes had little association with Rab5 early endosomes (Fig. 6d, e). However, as Rab35 compartments grew in size and matured in lifetime, small early endosomes often approached Rab35 compartments and had brief associations at the periphery of a Rab35 compartment (Fig. 6d, e). These Rab5 early endosomes appeared to grow in size before rapidly moving away from Rab35 compartments (Fig. 6d). These results are consistent with Rab35 functioning to feed endocytosed membrane and protein components to early endosomes, thus decreasing available AP plasma membrane during interfacial contraction.

### Rab35 functions upstream of junction-associated Myosin II

**Fig. 7:**
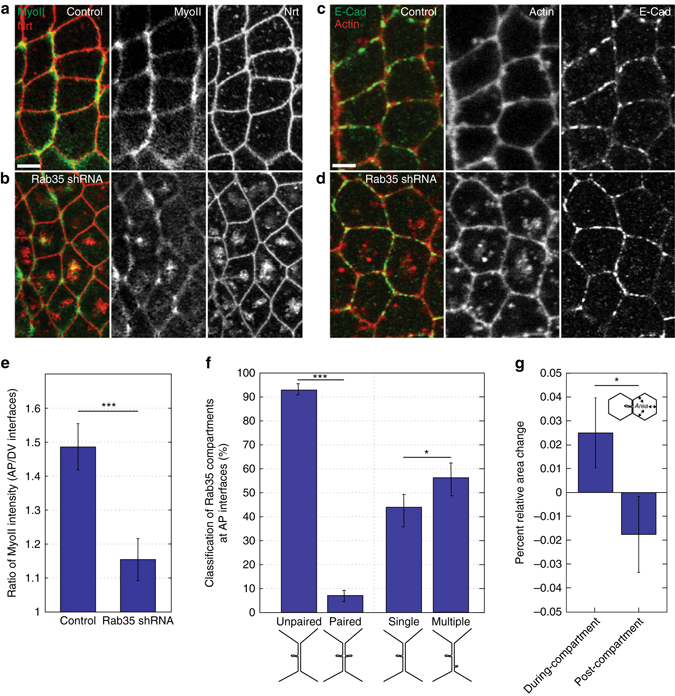
Rab35 compartments demonstrate interfacial monopolarity and are essential for planar polarized junctional actomyosin networks. **a**–**d** Disruption of Rab35 function leads to cytoplasmic aggregation of a plasma membrane protein (anti-Nrt, *red*, **a**, **b**), Myosin II (anti-Zipper, *green*, **a**, **b**), and F-actin (phalloidin, *red*, **c**, **d**). Anti-E-cadherin is in *green* (**c**, **d**). **a**, **c** Control (CRISPR:GFP:Rab35) or **b**, **d** Rab35-disrupted (CRISPR:GFP:Rab35; GFP shRNA) embryos. **e** Junctional Myosin II planar polarity is disrupted when Rab35 function is compromised (quantification of AP/DV junctional Myosin II ratios in CRISPR:GFP:Rab35 or CRISPR:GFP:Rab35; GFP shRNA-expressing embryos, *n* = 143 and 148 cells, respectively). **f** Rab35 compartmental behaviors suggest asymmetric force generation. Frequency of unpaired/paired and single/multiple compartments at single interfaces (*n* = 200 interfaces). **g** Coupling of area oscillations and Rab35 compartment behaviors. Apical area contraction in the opposing cell occurs at the termination of a Rab35 compartment. Student’s *t*-test (**e**, **g**), Mann–Whitney U Test (**f**), *P < 0.05, **P < 0.005, ***P < 0.0005. *Error bars* indicate standard error. *Scale bars* in **a**, **c** are 2.5 microns

We then examined trafficking and Myosin II dynamics in Rab35 compromised embryos. Interestingly, Neurotactin, an integral plasma membrane protein, accumulates in the apical cytoplasm (Fig. 7b), consistent with a block in membrane trafficking in CRISPR GFP:Rab35; GFP shRNAi embryos (91% of cells possess cytoplasmic Nrt aggregates, *n* = 218 cells). Myosin II also accumulates at these sites (86% of Nrt aggregates mislocalize Myosin II to these same structures, and junctional Myosin II populations are depleted or absent; Fig. 7b). Myosin II planar polarity is reduced in Rab35-disrupted embryos (Fig. 7e), and F-actin cytoplasmic aggregates occur as well (Fig. 7d). These results are consistent with an upstream function of Rab35 in triggering a Myosin II scissioning activity.

### Evidence for interfacial monopolarity during intercalation

In examining Rab35 compartmental behaviors, it was immediately apparent that paired Rab35 compartments are rare at individual interfaces (Fig. 1a, b). Indeed, only 7% of compartments had an identically paired compartment on the opposing surface, and 47% of AP Rab35 compartments had no compartment form at any point on the other side of the interface during the lifetime of the compartment (Fig. 7f). Given the above relationship between Rab35 and Myosin II, this suggests that interfaces may experience unipolar force generation, and would be consistent with the generation of interfacial shear forces^42^. To study these asymmetries more closely, we examined Rab35 compartmental behaviors and changes in apical cell area in the opposing cell. These results revealed that the opposing cell initiates apical area contractions after the termination of a Rab35 compartment (Fig. 7g), indicating a mechanical or signaling-based coordination of behaviors between cells that share a contracting AP interface.

### Rab35 functions in a conserved cell-shaping mechanism

**Fig. 8:**
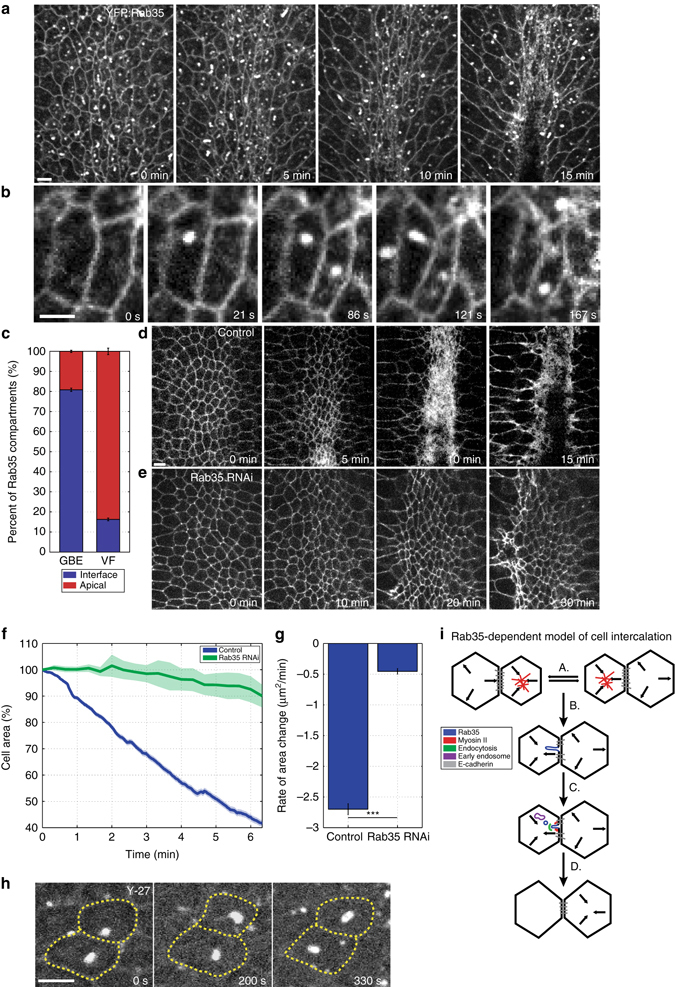
Rab35 compartments represent a common contractile mechanism. **a** Rab35 compartments form at contractile surfaces in cells undergoing apical constriction. Time-lapse images of an embryo expressing YFP:Rab35 during ventral furrow formation. **b**, **c** Rab35 compartments form on the medial-apical cell surface away from cell–cell interfaces. **c** Rab35 compartments display radial cell polarity during apical constriction (*n* = 261 compartments, *P* < 0.005). **d**, **e** Still frames of embryo labeled with a cell outline marker injected with control Rh3 (**d**) or Rab35 dsRNA (**e**) during ventral furrow formation. After 15 min the control embryo has undergone complete furrow formation (**d**), whereas in the Rab35 knockdown embryo apical constriction displays a frozen phenotype and furrow formation does not occur even after 30 min (**e**). **f** Plot of percent apical area contraction from automated analysis of ventral furrow cells in control (*blue*, *n* = 226 cells) and Rab35 (*green*, *n* = 313 cells) dsRNA embryos. **g** Reduction in area rate changes and decreased apical contraction in Rab35 dsRNA embryos compared to control embryos. **h** CRISPR GFP:Rab35-labeled embryos injected with Y-27632 Rok inhibitor during ventral furrow formation. **i** Model of Rab35-driven cell shape changes during cell intercalation. (A) Cells undergo oscillatory periods of apical area constriction driven by medial Myosin II (*red*). (B) Rab35 (*blue*) compartments take up excess plasma membrane during periods of asymmetric tension generation. (C) Rab35 directs compartment termination through the activity of junctional Myosin II (*red*) and endocytosis (*green*) with membrane delivery to internal endosomal stores (*purple*). (D) AP interfaces are shortened by the uptake of plasma membrane and/or E-cadherin adhesion complexes (*gray*), preventing a reversal in interface length. Anterior is up and ventral surface is presented in all images. Student’s *t*-test, ***P < 0.0005. *Error bars* indicate standard error. *Scale bars* for **a**, **b** are 5 microns and for **d**, **h** are 2.5 microns

Finally, we asked if Rab35 directs a common contractile mechanism involved in the generation of cell shapes. The invagination of mesoderm in the early *Drosophila* embryo occurs through the formation of a ventrally located furrow^43, 44^. Furrow formation is driven by constriction of the apical epithelial surface, a process that relies on radially polarized actomyosin behaviors^45–48^. Here, too, we found that Rab35 displays compartmental behaviors at the cell surface. Rab35 compartments form at the apical end of cells undergoing apical constriction in the ventral furrow (Fig. 8a). However, Rab35 compartmental behaviors, while polarized in the apical–basal axis, do not demonstrate planar polarities (Fig. 8b) and are enriched apically and not at interfaces (Fig. 8c). Rab35 compartments are therefore specifically polarized to shrinking cell surfaces in cells undergoing cell intercalation and apical constriction in the early embryo. Rab35 function is required for mesoderm invagination, as ventral furrow formation fails in embryos injected with Rab35 dsRNA (Fig. 8e). Additionally, rates of apical constriction are greatly reduced in embryos in which Rab35 function has been compromised (Fig. 8f, g), and the formation of apical Rab35 compartments displays a similar Myosin II independence (Fig. 8h). These results reveal a common direction of Rab35 to shrinking cell surfaces as well as a requirement for Rab35 function in shaping epithelial cell behaviors during development.

## Discussion

The generation of organismal and tissue shape is one of the most fundamental events that multicellular organisms must accomplish. Previous models of cell intercalation have focused on actomyosin-driven behaviors; however, the trafficking networks that are functioning in these cells, and the degree to which their activities may be coordinated with the cytoskeletal forces that control plasma membrane topologies, has been unclear. Additionally, assemblies of actomyosin networks are transient, but the effects of their function on cell shape must be maintained after force exertion. Our results have shown that a trafficking network centered on Rab35 acts as a ratcheting mechanism to ensure that interface contraction is progressive and irreversible. Rab35 functions upstream of junctional actomyosin forces during interface contraction to provide a membranous ratchet, and suggests that cytoskeletal-derived forces are required to terminate compartmental behaviors. Rab35 compartments are more numerous and possess longer compartmental times at contractile interfaces of actively intercalating cells. Rab35 compartments form at the plasma membrane and rapidly grow in size during periods of interface contraction, before shrinking through endocytic-dependent processes. Rab35 compartmental behaviors therefore represent a critical point of convergence at which cytoskeletal and membrane trafficking pathways function to drive changes in cell shape.

Changes in cell shape in many systems are driven by pulsatile processes that initiate directed movement before cycling through periods in which the force generating network reforms^14–16, 47, 49^. Previous work on ratcheting function has concentrated on different actomyosin regimes governed by Myosin II regulators such as Rho1 and Rok that trigger contraction and cell ratcheting^39^. Importantly, when Rab35 function is disrupted, apical cell areas maintain oscillatory behaviors and AP interface lengths still undergo brief periods of contraction. However, contractile periods are followed by reversals and interfaces re-lengthen, producing a failure in interface shortening. This “wobble” behavior is consistent with Rab35 functioning as a ratchet ensuring unidirectional movement during interface contraction. These results also suggest that junctional actomyosin-driven behaviors may be responding to an upstream trafficking apparatus centered on Rab35. Indeed, junctional Myosin II localization is disrupted in Rab35 knockdown embryos, and accumulates intracellularly along with integral membrane proteins (Neurotactin) and F-actin. In tissue culture cells, it has been shown that disruption of Rab35 similarly leads to an accumulation of F-actin during abscission, which provides another link between Rab35 and cytoskeletal dynamics^33, 35^. Finally, and again consistent with expectations for a compartment-driven ratcheting function, it is also striking that Rab35 compartments are not present uniformly during cell intercalation, but begin to form specifically as interface contraction occurs.

The AP patterning system is responsible for directing Rab35 compartment formation away from apical/medial sites and functionally engaging Rab35 at AP interfaces. It is interesting to note that high levels of anterior patterning information, such as is present in the head region, inhibit planar polarity, cell intercalation, and Rab35 compartment dynamics, while maintaining interfacial Rab35. The common appearance of apically localized Rab35 compartments in the ventral furrow and in *bnt* mutant embryos suggests a model in which ventral fate genes could mediate apical constriction by re-directing Rab35 compartment formation through the inhibition of AP patterning cues.

Myosin II is found in several different populations within intercalating cells. Transient, web-like Myosin II localizations are found in highly dynamic structures in the apical/medial regions of epithelial cells, while more stable, cable-like structures are present at cell junctions^9, 10, 15, 16^. Junctional Myosin II enrichment is dependent on Rab35 function, and is required for the termination of Rab35 compartments. However, the role of apical/medial actomyosin forces and cell oscillations on Rab35 compartmental behaviors is less clear. It appears that Rab35 compartments may require Myosin II medial “flows”^15^ to function as a polarizing cue, as Rab35 compartments become less planar polarized in the absence of Myosin II function. We hypothesize that cell oscillations produce cycles of high and low tension during which Rab35 compartments can form as infoldings of slackened plasma membrane that, when internalized, prevents interface length from rebounding to the same length as was present prior to area contraction (Fig. 8i). It is important to note that the termination of Rab35 compartments and the internalization of membrane is critical to ratcheting and productive interface contraction, as merely driving more plasma membrane into Rab35 compartments (as is observed in Y-27632, PitStop2, or chlorpromazine-injected embryos) in the absence of compartment termination does not direct lasting changes in cell topologies.

These results also suggest how oscillatory area contractions can be linked to productive movements. In worm and fly embryos^14, 47^, actomyosin contractions show both productive and non-productive periods. The functional engagement of area oscillations may depend on the presence of the Rab35 ratchet, as immediately prior to intercalation Rab35 compartments are absent from cell interfaces (Fig. 1a). However, it is intriguing that Rab35 compartments can form when Myosin II function is compromised in Y-27632-injected embryos. It may be that the small amount of Myosin II function left after Y-27632 injection is enough to drive small oscillations in area, or that thermal fluctuations in cell area are enough for compartment formation. Regardless, this will be an interesting area for further studies.

What is the nature of Rab35 compartments? While there are punctate Rab35 compartments present in the apical cytoplasm that partially colocalize with endosomal markers, the major population of Rab35 during early gastrulation and cell intercalation is present in tubular compartments that are contiguous with the cell surface. The extremely elongated Rab35 plasma membrane tubules observed in either Myosin II-disrupted or endocytosis-inhibited embryos, as well as the immediate filling of Rab35 compartments at the cell surface, are consistent with Rab35 marking endocytic infoldings of the plasma membrane. Additionally, Rab35 compartments are positive for a plasma membrane PIP_2_ species, PtdIns(4,5)P_2_, and Rab35 immunogold TEM imaging and the quantification of immunogold particle localization demonstrates that Rab35 is largely present in compartments at the cell surface. Indeed, open infoldings marked by Rab35 immunogold form in subapical zones near the adherens junctions. It is interesting to note that similar open, tubular structures have also been observed by TEM near electron dense junctions during cell intercalation^18^. When Rab35 function is compromised, there is a shift of endocytic, dextran uptake away from AP interfaces and a dramatic increase in failed endocytic events. In tissue culture, Rab35 has been shown to function early in endosomal pathways and immediately after vesicle scission, to drive the generation of newborn endosomes^37^. Our data suggest a subtle shift in Rab35 function to an earlier time point occurs during early *Drosophila* morphogenesis, but is broadly consistent with a Rab35-driven function in directing the delivery of membrane from the cell surface to endosomal pathways. However, our results do not exclude an added role for Rab35 at endosomal compartments, as F-actin and Myosin II accumulate intracellularly in Rab35 compromised embryos. This accumulation is at substantially lower levels than occurs at the cell cortex in wild-type embryos, but could contribute to the observed cell intercalation dynamics, especially the ~17% decrease in oscillatory area amplitudes.

We also examined the association of Rab35 compartments with markers for early (Rab5) or recycling (Rab11) endosomes. While the large majority of Rab35 compartments do not localize with early or recycling endosomes, there was a small fraction of compartments that do (15%). Interestingly, when observed under live imaging conditions, it became apparent that Rab35 compartments do often have transient interactions with endosomal compartments (98%) that occur specifically toward the end of Rab35 lifetimes, consistent with Rab35 feeding endocytosed membrane through a specialized plasma membrane contiguous compartment. These results are consistent with the broader conclusion of our work that endocytic uptake of plasma membrane components is essential to ensuring irreversible changes in interface length that drive cell neighbor exchange. This endocytic uptake could function through two potential mechanisms: (1) E-cadherin adhesion complex removal, or (2) general membrane removal, which would require that individual interfaces possess isolated membrane domains. Embryos that expressed fluorescently labeled Rab35 and E-cadherin did not express E-cadherin at levels sufficient to be resolvable by confocal microscopy, thus making it difficult to distinguish between these models, but this will be an important open question going forward. Previous work has proposed that MyoII function clusters E-cad complexes for endocytosis^18^, while our results show that Rab35 functions upstream of junctional Myosin. Thus, Rab35 compartments may serve to direct Myosin II clustering of E-cadherin adhesion complexes for endocytosis and junctional remodeling.

An interesting feature of Rab35 behaviors is the absence of paired compartments at contracting interfaces. Approximately 50% of Rab35 compartments form without the presence of any Rab35 compartments on the opposing side of an interface, and 93% of compartments are present without a matching, synchronous Rab35 compartment. This monopolarity of Rab35 function is the first evidence of asymmetric behaviors across a common, shared interface during early GBE. As Rab35 compartmental behaviors reflect Myosin II activity, this further suggests that there are likely anisotropic forces on opposing sides of individual interfaces. This anisotropy could drive the generation of shear forces that would cause the dissociation of homophilic extracellular E-cadherin bonds^42^. It is also intriguing that, in terms of area oscillations, Rab35 compartmental formation is best correlated with cycles of area contractions in the opposing cell at a shared interface. This would be consistent with the above model in which changes in apical cell area are transmitted through E-cadherin junctions to a neighboring cell that may be in a differentially tensioned state (Fig. 8i). This may then again permit slackened plasma membrane to be taken up by Rab35-dependent endocytic processes.

## Methods

### Construction of the CRISPR GFP:Rab35 transgenic line

To create transgenic lines of CRISPR GFP:Rab35, a homology-directed repair (HDR) donor plasmid was generated comprising the genomic DNA 2.5 kb upstream of the *Rab35* ATG start site cloned into pCasper4 plasmid (*Drosophila* Genomics Resource Center, stock #1213), followed by the coding sequence of GFP. This upstream Rab35 and GFP sequence were digested out of pCasper and cloned into pBluescript KS (Addgene). Then, the final 3.5 kb downstream of the *Rab35* ATG start site was subsequently cloned into pBluescript KS. In total, 6 kb of the genomic *Rab35* locus was cloned and inserted into pBluescript KS along with the 717 bp coding sequence of eGFP, using standard PCR and cloning protocols to create the HDR donor plasmid. Two gRNA sequences that serve to guide the Cas9 nuclease to specific genomic locations were designed according to Gokcezade et al.^50^ and inserted into pU6-BbsI-gRNA (AddGene stock #45946). All constructs were sequence verified (UC Denver Core Sequencing). gRNAs (Supplementary Table 1; 100 ng/µl) and GFP:Rab35 plasmid (150 ng/µl) were co-injected into Cas9-expressing flies (*y* ^*2*^ *cho* ^2^ *v* ^*1*^; *P{nos-Cas9*, *y* *+* , *v* *+* *}3* *A/TM6C*, *Sb Tb*; CAS-0003) by BestGene. Surviving adults were crossed to *w* ^*lethal*^ */FM7*; *Sb/Tm3*, *Ser* to create possible insertion lines. Embryos from these individual lines were tested for fluorescence using a spinning disk confocal microscope, and insertion into the genome was verified by PCR (see below). Successful insertions were relatively rare: 200 lines were scored before recovery of CRISPR GFP:Rab35. Cloning primers for HDR donor GFP:Rab35 are listed in Supplementary Table 1.

### PCR verification of the CRISPR GFP:Rab35 transgenic line

PCR primers (see Supplementary Table 1) to verify correct insertion of GFP into the *Drosophila* genome via CRISPR were designed in the following manner. The 5′ primer was targeted against a genomic region outside of that which was cloned into the HDR donor GFP:Rab35 plasmid (2.6 kb upstream of *Rab35* start codon), while the 3′ primer was targeted against a region within the GFP coding sequence. Thus, a PCR product could only be produced when eGFP has been integrated into the genomic *Rab35* locus. Three primer pairs were designed in this manner (NIH, Primer-BLAST) and PCR was performed in both CRISPR GFP:Rab35 flies and control OreR flies.

### Confocal microscopy and time-lapse imaging

Confocal images were acquired on an Olympus Fluoview FV1000 confocal laser scanning microscope with 40×/1.35 NA or 60×/1.42 NA objective for fixed specimens. Time-lapse imaging was performed on a CSU10b Yokogawa spinning disk confocal from Zeiss and Solamere Technologies Group with 63×/1.4 NA objective. Embryos were imaged after dechorionation and placement on a gas-permeable membrane in Halocarbon 27 oil. Live imaging was performed using exposure settings of 100–350 ms and images were acquired every 1 s.

### Embryo fixation and immunostaining

Embryos were dechorionated in 50% bleach solution and fixed for 1 h 15 min at the interface of heptane and 3.7% formaldehyde in 0.1 M sodium phosphate buffer (pH 7.4) before being manually devitellinized (or heat fixed and devitellinized by osmotic shock for staining with anti-zipper) and stained with Alexa-546 phalloidin (1:200; Molecular Probes), Rat anti-DE-cadherin (1:100; DSHB), Mouse anti-Neurotactin (1:1; DSHB), Rabbit anti-Zipper (1:100; gift from E. Wieschaus Lab), Rabbit anti-Rab11 (1:100, gift from D. Ready Lab), Rabbit anti-Rab5 (1:100, gift from M. Zerial Lab). Conjugated secondary antibodies Alexa-488 and Alexa-568 (1:500; Molecular Probes) were used. Embryos were mounted in Prolong Gold with DAPI (Molecular Probes).

### Embryo processing for immunogold TEM

Endogenous CRISPR:GFP:Rab35 embryos were dechorionated in 50% bleach solution and fixed for 45 min at the interface of heptane and 16% formaldehyde in 50 mM sodium cacodylate buffer (pH 7.4) before being manually devitellinized. Embryos were stained with anti-GFP antibody and 6 nm gold-conjugated anti-mouse antibody before embedding. Embryos were then post-fixed in a solution containing 1% OsO_4_ in 50 mM cacodylate buffer (pH 7.4). Embryos were imaged on a FEI Tecnai G2 Biotwin Transmission Electron Microscope, run at 80 kV.

### Automated time-lapse imaging analysis

Image and data analysis were performed in Matlab. Cells were segmented using a seeded watershed algorithm and tracked in time. The “skeletonized” representation of the tissue directly yields vertex positions, interface contours, lengths and orientation angles, cell areas and perimeters, which we store together with cell–cell and vertex–vertex connectivity matrices. We measured cell areas as the sum of the pixels within the contour of the watershed segmentation lines (multiplied by the pixel area). Interface lengths were calculated as the Euclidian distances between the corresponding vertices.

*Fitting mean squared displacement (MSD) curves*. The MSD for the length is defined as $MSD(\tau ) = \frac{1}{{N - n}}\mathop {\sum}\nolimits_{k = 1}^{N - n} {{{\left[ {l((k + n)\partial t) - l(k\partial t)} \right]}^2}}$, where the time lag is $\tau = n \cdot \partial t$, *n* is the number of frames for the time shift, $\partial t$ is the time between frames, and *N* is the total number of available time points for the length trajectories *l*. We fit MSD curves to the function $MSD(\tau ) = A \cdot (1 - \exp ( - \alpha \tau )) + 2 \cdot B \cdot \tau + {C^2} \cdot {\tau ^2}$, where the first term represents the constrained component with amplitude *A* and positive time constant *α*, the second term is the diffusive term with diffusion coefficient *B*, and the third term the active component with *C* representing the velocity. We fit our data to this function using a least squares fitting method.

*Step detection*. To detect active motion steps in our vertex position trajectories we used a rolling analysis window technique adapted from Huet et al.^51^ The MSD is the customary method to classify a sub-trajectory into active, diffusive, or constrained motions based on whether the MSD curves upward, is linear, or curves downward, respectively. For periods of active motion the MSD behaves as a power law $MSD(\tau ) \propto {\tau ^\gamma }$, $MSD(\tau ) \propto {\tau ^\gamma }$ where *γ *> 1. By calculating the parameter gamma along a trajectory using a rolling window we can identify periods of active and non-active (i.e., either diffusive or constrained) motion. For each window we fit the MSD to lags between 5 and 3(*N*-1)/4, where *N* is the odd-numbered number of points in the window. The first 4 lags were left out of the fitting because localization error leads to artifactual subdiffusion at this short-time scale lowering the value of *γ*. To reduce computation time we performed linear fitting of the MSD verses *τ* on a log–log plot. Prior to step detection, trajectories were filtered with a 5th-order median filter using the *medfilt1* function in Matlab to remove noise.

One of the challenges of a rolling analysis window is in selecting the size of the window. A window too large makes detecting brief steps unlikely, whereas a window too short performs worse at detecting longer steps. Thus, we used a rolling window with variable widths optimized for the steps in our trajectories. For a given position of the window, which is defined by its center position, *γ* is calculated for a range of windows from *W*_min_ to *W*_max_ (we used the windows 21, 41, 61, and 81 frames) and the maximum value of *γ* is retained, corresponding to maximum likelihood of systematic motion. This is repeated for each time point along the trajectory to give *γ* as a function of time. The determination of systematic from non-systematic periods is made by setting a threshold on *γ*(*t*) of 1. We applied a minimum duration requirement of 14 frames (14 s) because we found that positive detections below that duration did not represent real-active periods.

*Neighbor number distributions*. We measured the number of cell neighbors for each segmented cell in every frame of the movie. Cells that touched the background (i.e., the edge of the segmented region) were excluded because their total neighbor number was unknown. For each frame the fraction of cells of each neighbor number was computed. We plotted the fractions of cells with 4, 5, 6, 7, and 8 sides. Cells with 3 sides or more than 8 did occur at rare times in the movies but generally did not exceed 5% in number so their traces were excluded. The traces were Gaussian-smoothed using a sigma of 5 s.

*Amplitude of cell area oscillations*. To measure the amplitude of cell area oscillations we used a method based on the Hilbert transform, described in Hsu et al.^52^ that outputs the instantaneous amplitude, frequency, and phase information of a signal given that the noise-associated oscillations have been filtered out. We filtered the cell area trajectories using a Savitzky–Golay filter of polynomial order 3 and frame size 81 (Matlab function *sgolayfilt*). We also detrended the cell area signal by subtracting from it a long-timescale Gaussian filtered (sigma = 200 s) version of itself. This long-timescale filtered signal served as the local average area, which we used to compute the amplitude as a percentage of the cell area at each instant. For each cell we averaged the instantaneous amplitude percentage over its entire time course.

*Particle detection*. Particle detection was performed based on correlations of thresholded wavelet planes obtained from wavelet decomposition of raw image, a method proposed by Olivio-Martin^53^. This method works by determining various wavelet planes (*W*_*i*_(*x*, *y*), *i*=1, 2, …, *J*) (each wavelet plane emphasizing image features of different sizes) obtained by performing B3-spline approximation of *à-trous* wavelet transform on the raw image. Each of the wavelet planes determined is thresholded to extract the pixels above standard deviation of background of that plane. The wavelet planes are then multiplied to obtain a correlation image *P*, where $P(x,y) = \mathop {\prod}\nolimits_{i = 1}^J {{W_i}(x,y)} .$

A user defined thresholding filter is applied on the correlation image to obtain a noise suppressed image *S*(*x*, *y*). Local average (*E*_[9x9]_) and variance (*Var*_[9x9]_) (over 9 × 9 neighborhood) and global average (*E*) for each pixel in the *S*(*x*,*y*) are calculated and the noise suppressed image is further thresholded, i.e., a pixel in *S*(*x*,*y*) is considered to be part of a Rab35 particle if: *S*(*x*,*y*) > *E* and *S*(*x*,*y*) > *E*_[9x9]_ + 0.5 × *Var*_[9x9]_.

*Particle tracking*. Particle detection and tracking were performed as in Loerke et al.^54^ first, Rab35 compartments were detected using an *à-trous* wavelet transform decomposition of the image^53^, after which the detected particles were linked in time using a global optimization particle tracking approach^55^, which also performs gap closing. In this particular case, the gaps between two trajectories were closed if the positions of the trajectory “stubs” were within 4 pixels (0.66 µm) distance from each other and if the time gap was not more than 3 s.

*Association analysis*. A Rab35 compartment is considered to be uniquely associated with a specific interface if it satisfies all of the following conditions:

1. The Rab35 compartment is either on or within 10 pixels (1.66 µm) of the interface.
2. The Rab35 compartment is closer to this interface than to any other interfaces.
3. The Rab35 compartment is at least 1 pixel or 0.1 × length of the interface (whichever is less) away from the closest vertex.

*Density analysis*. The density of Rab35 particles at a time point *t* is given by $Density(t) = {N_{Rab35}}(t)/{N_{Interfaces}}(t),$ where *N*_*Rab*35_(*t*) is the number of Rab35 particles associated with interfaces of angle *θ* at time *t* and *N*_Interfaces_(*t*) is the number of interfaces of angle *θ* at time *t*.

*Length and area analysis*. The rate of interface length changes during compartment association are given by $\Delta L/\Delta t = \left[ {L({t_{end}}) - L({t_{start}})} \right]/({t_{end}} - {t_{start}})$, where *L* is the length of the interface, *t*_end_ is the time at the end of compartment association, and *t*_start_ is the time at the start of compartment association.

Area changes are calculated analogously, where the rate of area change is normalized to the average area of the cells over the considered period (yielding a unitless number).

Lifetime = (*t*_end_−*t*_start_) + 1, where *t*_start_ is the time frame where the Rab35 compartment becomes associated with an interface, *t*_end_ is the time frame after which the Rab35 compartment becomes disassociated with an interface.

*Classification of interfaces*. Interfaces are classified as either AP or transverse interfaces based on their initial angle (angle during the first 90 s of GBE). The interfaces with an angle of ±15° of the DV axis direction are considered AP interfaces, and the rest are considered transverse interfaces.

*Individual lifetime analysis*. Lifetime distributions of the Rab35 particles were corrected for movie length as in Loerke et al.^54^. Alternatively, due to signal noise and movement in *z*-axis, compartment lifetime averages were measured in ImageJ (Fig. 1d, g, Fig. 4c).

*Ventral furrow cell area analysis*. For the cell area verses time plot (Fig. 8f) each cell’s area trajectory was normalized such that at *t* = 0 the area was 100% and then took the average and standard error over all cells. For the rate of area change plot (Fig. 8g) for each cell we took the difference between the final and initial area over the full time period (6.6 min) divided by the time period and then took the average and standard error over all cells.

### siRNA preparation

Primers for siRNA treatments were chosen through the SNAPDRAGON RNAi design program that bioinformatically selects against off-target effects (*Drosophila* RNAi Screening Center). dsRNA was made using a Megascript T7 Transcription Kit (Ambion) and purified using Qiagen RNeasy columns. Final concentration was determined with a NanoDrop ND1000 spectrophotometer.

### RNAi treatments

Embryos were prepared in the same manner as for live imaging and then glued to a coverslip using heptane glue. Embryos were dehydrated for ~9 min, and then injected with siRNA against *Rh3* (26 µM), *Rab35* (288 bp, 52 µM). Embryos sat for 20–30 min prior to imaging for RNAi to take effect.

### Drug treatments and dyes

The following drugs were injected into embryos at a final concentration of 1:50 from the given stock solution: Pitstop2 (Abcam at 1.25 mM), Chlorpromazine (Sigma at 10 mM), Y-27632 (Tocris at 100 mM), LatB (Santa Cruz at 10 mM), Dextran Rhodamine B (ThermoFisher at 1 mg/mL). Embryos were imaged immediately following injection unless otherwise noted.

### Embryo scoring, analysis, and statistics

Embryos were collected on apple juice agar plates, and development was followed and scored under Halocarbon 27 oil on a Stemi 2000 Zeiss dissecting microscope with transillumination. Pooled data from multiple trials were summed, and RNAi scoring data were tested for statistical significance using a two-dimensional contingency table with a *χ* ^2^ test with *α* = 0.05. Tests were performed in two ways: (1) all data were tested to determine if changes in the proportions of phenotypes seen were significant, which resulted in *P* < 0.0001; (2) both RNAi treatment groups were tested against the *Rh3* control treatment to determine if the changes in the proportions of phenotypes were significant in relation to control proportions.

Nonparametric statistics were performed by Mann–Whitney U Test (Figs. 4b, c, 7f, 8c).

All *error bars* indicate measured standard error.

### Fixed and time-lapse image editing and quantification

Confocal and spinning disk images were edited using ImageJ and Adobe Photoshop. Channels for fixed images were uniformly leveled for optimal channel appearance. Time-lapse images were leveled with ImageJ or Photoshop to show optimal protein populations, or were processed with identical settings to permit comparisons of protein levels. Manual quantification of endogenous immunogold-labeled Rab35 images at 49,000× was performed by counting of gold particles in individual sections. Gold particles within 20 nm of apical surface or interface were assigned as apical surface-associated or interface-associated compartments, respectively. Gold particles within 20 nm of vesicles or tubular structures were assigned as labeling compartments. The quantity of gold particle localization at different places was expressed as the percentage of the total number of gold particles.

Manual colocalization between Rab35 and Rab5 or Rab11 was performed using Adobe Photoshop on live and fixed embryos. Quantification was performed by selecting puncta between 2 and 15 pixels (Olympus LSM 510 laser scanning confocal (fixed); pixel size = 0.056 µm/pixel) in size in set areas of 5 µm × 5 µm grids. The selected puncta were then overlaid with the opposing channel. When the overlapping region was larger than 2 pixels × 2 pixels, the relationship between two proteins was assigned as “colocalized”. Average colocalization was found by performing a weighted average calculation from a minimum of six individual images collected from three different embryos and standard deviations were plotted.

Manual quantification of association between Rab35 and Rab5 was performed using ImageJ on embryos that were imaged live. Rab35 puncta larger than 1 pixel (Solamere spinning disk; pixel size = 0.164 µm/pixel) were selected. Then, the selected puncta were tracked to examine overlap with the opposing channel over a compartment’s lifetime. When the overlapping region was larger than 1 pixel × 1 pixel and the overlapping period was longer than 1 s, the relationship between two proteins was assigned as “associated”. Average association was calculated by performing a weighted average calculation of images from three different embryos.

### Fly stocks and genetics

Fly stocks: UASp-YFP:Rab35 9821; UASp-deGradFP 58740; UAS-GFP TRiP Valium 20 41555; UAS-YFP:Rab5 24616; UAS-YFP:Rab11 9790; WT SqhAX3/FM7 (all BDSC); Resille:GFP;^10^ Spider:GFP;^10^ sqh:GFP;^56^ sqh:mCh;^47^ *bicoid*, *nanos*, *torso-like*/TM3 (gift of E. Wieschaus). Flies were maintained at 25 °C by standard procedures. UAS transgenic flies were crossed to Rab35 > Gal4 Bloomington *Drosophila* Stock Center (BDSC 51599; Supplementary Figs. 1, 4) or matαTub-Gal4VP16 67C;15 (D. St. Johnson, Gurdon Institute, Cambridge, UK) maternal driver females and second-generation embryos were analyzed. CRISPR:GFP:Rab35; UAS-deGradFP or CRISPR:GFP:Rab35; UAS-GFP shRNA TRiP were crossed to matαTub-Gal4VP16 67C; CRISPR:GFP:Rab35 and second-generation embryos were analyzed.

### Repeatability

All measurements were quantified from a minimum of three embryos, and represented at least two individual trials.

### Data and coding availability

All MatLab coding and algorithms, as well as primary data, are freely available from the corresponding author upon reasonable request.

## Electronic supplementary material

Supplementary Information

Supplementary Movie 1

Supplementary Movie 2

Supplementary Movie 3
