# Supplementary material for: Planar polarized Rab35 functions as an oscillatory ratchet during cell intercalation in the Drosophila epithelium
Source: Nat Commun. 2017 Sep 7;8:476. doi: 10.1038/s41467-017-00553-0 (PMC5589913; doi:10.1038/s41467-017-00553-0)
Supplement: Supplementary file 1 — Supplementary Information [file 41467_2017_553_MOESM1_ESM.pdf]

Description of Supplementary Files

File Name: Supplementary Information
Description: Supplementary Figures and Supplementary Table

File Name: Supplementary Movie 1
Description: **Rab35 localizes to dynamic, membrane-associated compartments.** Time-lapse images of an embryo expressing YFP:Rab35 during germband extension. Rab35 localizes primarily to compartments at AP interfaces and at low levels to the plasma membrane. Images acquired every 1s for 10.0 min. Anterior is to the left, dorsal is up.

File Name: Supplementary Movie 2
Description: **Lack of ratcheting behaviors in Rab35 compromised embryos.** Transient interface contractions occur in Rab35 dsRNA injected embryos, but are followed by rapid reversals in interface length. Left panel shows a T1 AP interface (blue line) undergoing active contractile and lengthening periods. Right panel shows automated quantitation of interface length (blue line). The active periods of stepping (both positive and negative changes in interface length) are shaded in blue. Images acquired every 1s for 9.5 min. Anterior is to the left, dorsal is up.

File Name: Supplementary Movie 3
Description: **Inhibition of Myosin II function results in elongated, tubular Rab35 compartments that cannot terminate.** Time-lapse images of an embryo expressing YFP:Rab35 injected with Y-27632 during germband extension. In the absence of Myosin II function, Rab35 compartments still form at interfaces, but fail to terminate and grow into elongated, tubular structures. Images acquired every 1s for 10.5 min. Anterior is to the left, dorsal is up.

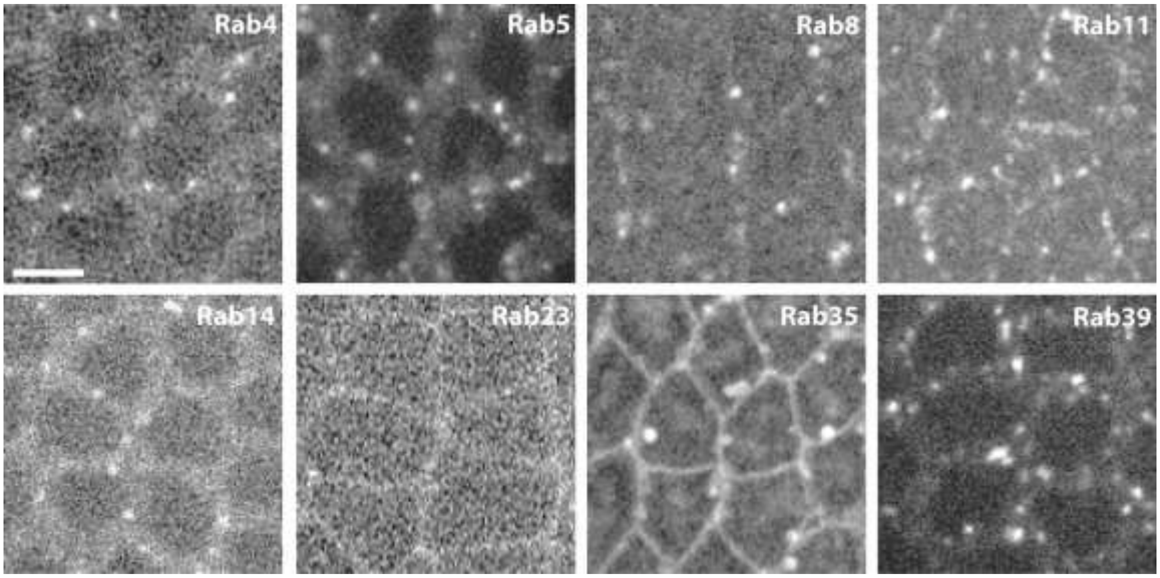

Supplementary Figure 1. Screen for Rab protein localization during germband extension. Still frames from live imaging of embryos expressing YFP-labeled Rab proteins. Rab4, 5, 8, 11, 14, 35, and 39 label punctate structures during cell rearrangements, whereas Rab23 shows cortical localization. Rab35 shows large interface-associated compartments and was therefore chosen for further analysis. Scale bar is 5 microns.

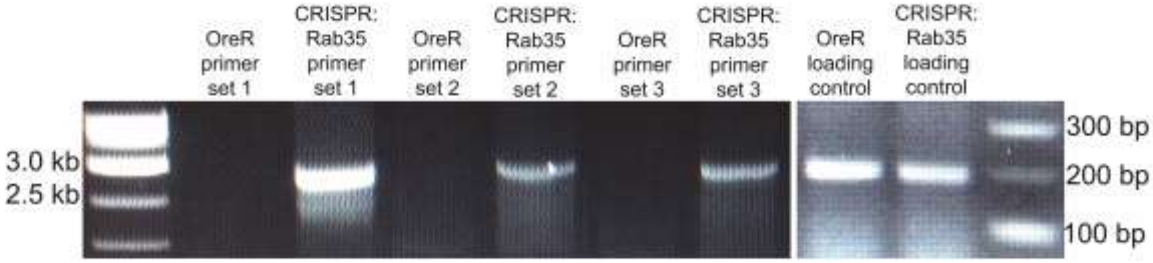

Supplementary Figure 2. Successful CRISPR-mediated insertion of GFP at the N-terminus of the endogenous Rab35 locus in *Drosophila*. Three primers pairs (set 1, 2, and 3) were designed such that the 5' primers bind upstream of the cloned homology donor plasmid and 3' primers bind to the GFP coding sequence. Successful amplification of these regions is observed only in CRISPR GFP:Rab35 embryos but not wild-type OreR control embryos, demonstrating that GFP correctly inserted at the N-terminus of endogenous Rab35. As a loading control, PCR of OreR and CRISPR:Rab35 genomic DNA was performed using primers located in the Rab35 coding sequence.

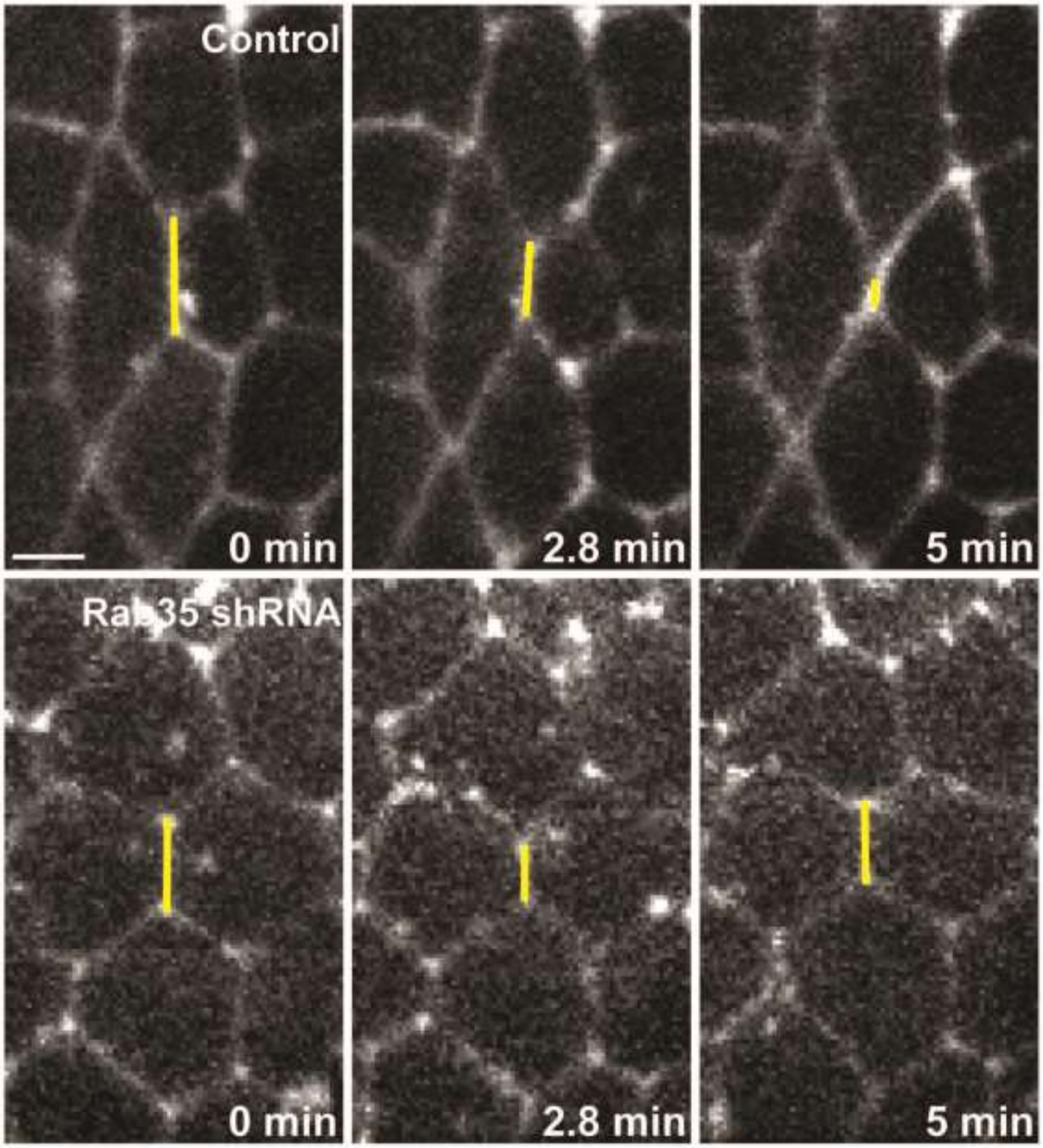

Supplementary Figure 3. Rab35 function is necessary for progressive interface contraction. Still frames from a CRISPR:GFP:Rab35 embryo (control) or CRISPR:GFP:Rab35; GFP shRNA embryo (Rab35 shRNA) injected with dextran to label cell outlines. In the control embryo (top panel) the interface outlined in yellow has fully contracted within 5 minutes, whereas in the Rab35 shRNA embryo (bottom panel) the interface begins to contract, but contraction is reversed resulting in no net interface contraction after 5 minutes. Scale bar is 2.5 microns.

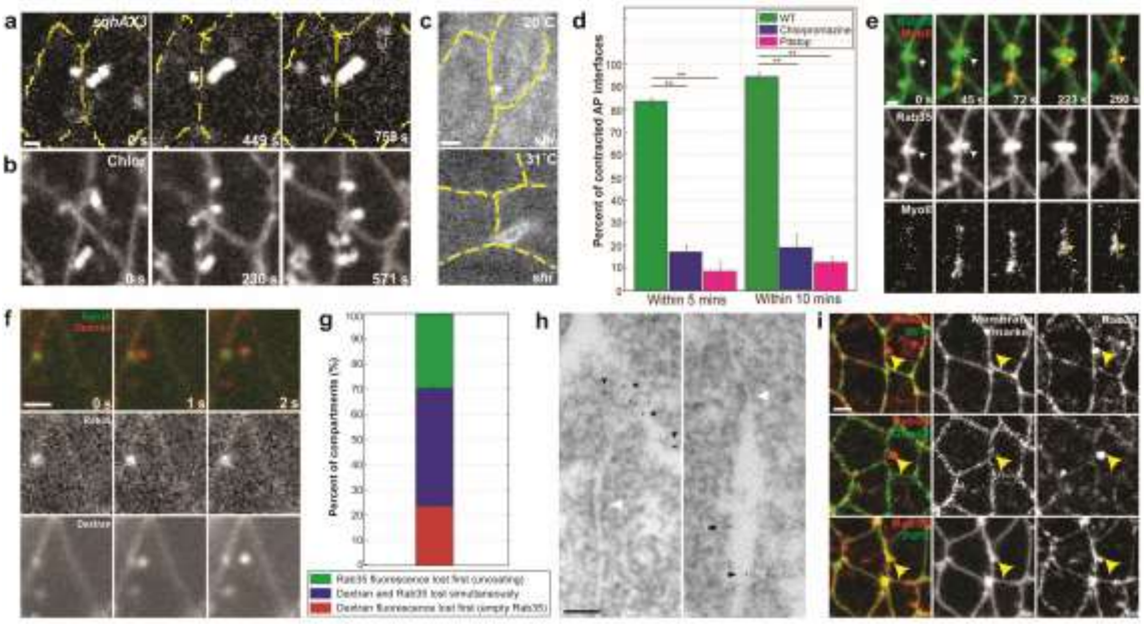

Supplementary Figure 4. Rab35 compartments require Myosin II and endocytic activity for termination, but form independent of their function. (a) Still frames of a CRISPR:GFP:Rab35, sqh^{AX3} hemizygous zygotically mutant embryo showing that Rab35 compartments form at cell interfaces (outlined in yellow-dashed lines) but fail to terminate in Myosin II mutant embryos. Zygotic mutant embryos did not show fully penetrant phenotypes (~60% penetrance), consistent with a partial maternal contribution to GBE. (b) YFP:Rab35 labeled embryo injected with endocytic inhibitor chlorpromazine. Compartments fail to resolve from cell interfaces even after 571 seconds. (c) Rab35 forms elongated tubular compartments when $\textit{shibire}^{ts1}$ is disrupted at the non-permissive temperature (31˚C). Due to *shi* and Rab35 both being located in the X chromosome, GFP:Rab35 is driven from a weaker sqh promoter transgene on chromosome III. (d) Disruption of AP interface contraction when endocytosis (chlorpromazine or PitStop2 injection) is compromised. (e) Rab35 compartments form at interfaces upstream of Myosin II junctional localization. Still frames of an embryo expressing homozygous YFP:Rab35, sqh:mCh, showing that Rab35 compartment formation precedes Myosin II appearance at interfaces (white arrowheads, 0s and 45s). Myosin II intensity is strongest during Rab35 compartment termination (yellow arrowhead compartment showing MyoII enrichment at 223s and 260s as compartment terminates). (f) Rab35 compartments serve as hubs for endocytosis. Still frames from a CRISPR:GFP:Rab35 embryo injected with dextran rhodamine. A dextran labeled vesicle buds off from a Rab35 compartment at the interface and moves into the intracellular space. (g) Quantitation of Rab35 and Dextran behaviors. 44% of the time Rab35 and dextran positive puncta detach from cell surface and then are lost simultaneously (blue, and Fig. 5i), 30% of the time a dextran positive puncta separates from a Rab35 compartment (green), and 24% of the time a Rab35 compartment loses dextran fluorescence (red)**.** (h) Two examples of Rab35 immunogold TEM, showing juxtaposition of adhernes junctions (white arrowhead) with Rab35 (black arrowheads). (i) Rab35 compartments show privileged membrane composition. Still frames from embryos expressing mCh:Rab35; 95-1 (top row), CRISPR:GFP:Rab35; Gap43:mCh (middle row), or mCh:Rab35; PLC:PH:GFP (bottom row). For consistency, Rab35 was artificially colored red and membrane markers colored green in images. 95-1 and Gap43 labeled interface shows no colocalization with Rab35 compartments (yellow arrowheads), whereas PLC:PH colocalizes with the Rab35 compartment. Error bars indicate standard error. Scale bars

in (a) and (c) are 1.25 microns, scale bars in (e) and (h) are 2.5 microns, scale bar in (f) is 2 microns, and scale bar in (i) is 100nm.

Supplementary Table 1

Primer	Sequence
5' Rab35 RNAi	TAATACGACTCACTATAGGGGGCTGGAGGAGATCCAGAAT
3' Rab35 RNAi	TAATACGACTCACTATAGGGTGTCTTTGGCAGATGTCTCG
5' Rh3 RNAi1	TAATACGACTCACTATAGGGCTGGTCATCAATCTGGCCTT
3' Rh3 RNAi1	TAATACGACTCACTATAGGGGGAGCCAATGATTCCAAAGA
5' Rh3 RNAi2	TAATACGACTCACTATAGGGGGATCAGGCCAAGAAGATGA
3' Rh3 RNAi2	TAATACGACTCACTATAGGGAGGAAGCATATGGTGATGGC
5' CRISPRverify1	ATGATGCCGTCTTTCAGCGTG
3' CRISPRverify1	ACCACCCCGGTGAACAGCTC
5' CRISPRverify2	TGATGCCGTCTTTCAGCGTGC
3' CRISPRverify2	ACCACCCCGGTGAACAGCTCC
5' CRISPRverify3	CCTCCGACGATGATGCCGTCT
3' CRISPRverify3	GTGAACAGCTCCTCGCCCTTGC
5' CRISPRgRNA1	CTTCGCACCTGGTCTGTGGGAAAC
3' CRISPRgRNA1	AAACGTTTCCCACAGACCAGGTGC
5' CRISPRgRNA2	CTTCGTGCTTTTATATTTACAGTT
3' CRISPRgRNA2	AAACAACTGTAAATATAAAAGCAC
5' HDR14500KpnI	GGGGTACCACCATCTACTTCTGCGGG
3' HDR16993EcoRI	GGGGGAATTCTTTTCGACGTTTTTTCGC

5’ HDRGFPEcoRI	AAAGGAATTCATGGTGAGCAAGGGCG
3’ HDRGFPBamHI	ATTGGATCCCTTGTACAGCTCGTCCATGC
3’ SacIIGFP	ACCGCGGCTTGTACAGCTCGTCCATG
5’ HDR16994SacII	ACCGCGGATGGCACGCGGCTT
3’ HDR20500BamHI	CAAGGATCCCTCGTGCTCTCCCCAAC
